# Condition-Dependent Coordination and Peroxidase Activity of Hemin-Aβ Complexes

**DOI:** 10.3390/molecules25215044

**Published:** 2020-10-30

**Authors:** Chiara Bacchella, James T. Brewster, Steffen Bähring, Simone Dell’Acqua, Harrison D. Root, Gregory D. Thiabaud, James F. Reuther, Enrico Monzani, Jonathan L. Sessler, Luigi Casella

**Affiliations:** 1 Department of Chemistry, University of Pavia, Via Taramelli 12, 27100 Pavia, Italy; chiara92_b@libero.it (C.B.); simone.dellacqua@unipv.it (S.D.); enrico.monzani@unipv.it (E.M.); 2 Department of Chemistry, The University of Texas at Austin, 105 East 24th, Street-Stop A5300, Austin, TX 78712-1224, USA; jbrewste@utexas.edu (J.T.B.II); hroot@utexas.edu (H.D.R.); gtamanaco@msn.com (G.D.T.); James_Reuther@uml.edu (J.F.R.); 3 Department of Physics, Chemistry, and Pharmacy, University of Southern Denmark, Campusvej 55, DK-5230 Odense, Denmark; 4 Department of Chemistry, University of Massachusetts Lowell, Lowell, MA 01854, USA

**Keywords:** central nervous system, neurodegeneration, Alzheimer’s disease, hemin, peroxidase

## Abstract

The peroxidase activity of hemin-peptide complexes remains a potential factor in oxidative damage relevant to neurodegeneration. Here, we present the effect of temperature, ionic strength, and pH relevant to pathophysiological conditions on the dynamic equilibrium between high-spin and low-spin hemin-Aβ_40_ constructs. This influence on peroxidase activity was also demonstrated using 2,2′-azino-bis(3-ethylbenzothiazoline-6-sulfonic acid) (ABTS) and dopamine (DA) oxidation rate analyses with increasing ratios of Aβ_16_ and Aβ_40_ (up to 100 equivalents). Interaction and reactivity studies of aggregated Aβ_40_-hemin revealed enhanced peroxidase activity versus hemin alone. Comparison of the results obtained using Aβ_16_ and Aβ_40_ amyloid beta peptides revealed marked differences and provide insight into the potential effects of hemin-Aβ on neurological disease progression.

## 1. Introduction

Central nervous system (CNS) neurodegeneration is characterized by the dysfunction and death of neurons in the brain and spinal cord. Recent efforts focused on elucidating the pathophysiological origins of CNS neurodegeneration, coupled with clinical failures on clearing amyloid beta constructs, suggests the need for an in-depth understanding of the etiology in order to design better treatment protocols [1,2,3]. Within this paradigm considerable effort remains focused on determining the roles that metal ions may play in the structural modification of protein aggregates, as well as aberrant oxidative and nitrative modification of various cellular components (e.g., DNA, cell membrane, proteins, and small molecules) [4,5,6,7].

The dysfunction of regulatory ferric heme (hemin) pathways present in neurodegenerative diseases, compounded by the interaction of hemin with amyloid beta (Aβ) and attendant peroxidase activity, has led to the proposal that hemin plays a role in disease progression [8,9,10]. Hemin has also been found to suppress Aβ-induced inflammatory activation of astrocytes and may retard other amyloid clearance pathways, further complicating etiological analyses [11,12]. A number of elegant studies by the groups of Casella [13], Atamna [14,15,16] Gao [17], Ghosh and Dey [18,19,20], Flemmig [21], Lu and Peng [22] and Imhof [23,24] have demonstrated the interaction between hemin and Aβ peptides (i.e., Aβ_16_, Aβ_40_, Aβ_42_, and mutant Aβ) with an increase in peroxidase activity [9]. Previous work has also led to the suggestion that at high concentrations of Aβ and hemin or high ratios (ca. 8 equivalents) of Aβ to hemin, a cytochrome *b*-type species predominates and can attenuate reactivity [20]. However, yet to be catalogued fully are the precise differences in coordination and peroxidase activity observed with Aβ_40_, a major amyloid species in diseased brains [25], at physiological temperature (37 °C), pH relevant to neuroinflammation (acidic pH), at high Aβ (i.e., 20 to 100 equivalents) to hemin ratios, and with aggregated Aβ. Here, we report a detailed condition-dependent modulation of the coordination environment and reactivity of hemin-Aβ constructs. As described below, under conditions designed to mimic those associated with neuroinflammation, such as at acidic pH (i.e., pH 6.3) [26,27] and at high ratios of Aβ-to-hemin that are potentially reflective of local concentrations in neuroinflammation or after traumatic brain injury (TBI) [21,28,29,30], the peroxidase activity of hemin-Aβ_40_ and Aβ_16_ complexes were significantly enhanced, as demonstrated via the oxidation of 2,2′-azino-bis(3-ethylbenzothiazoline-6-sulfonic acid) (ABTS) to ABTS radical cation and the oxidation of dopamine to dopaminochrome.

## 2. Results and Discussion

**Figure 1:**
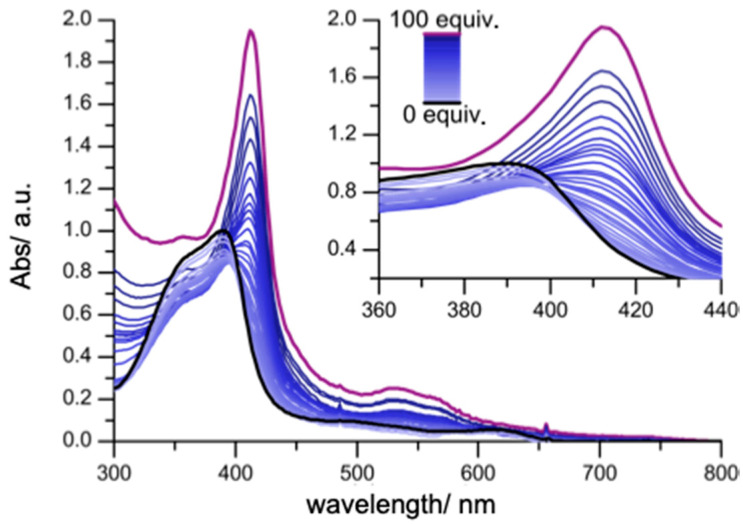
UV-vis spectrum of hemin (5 μM) upon addition of Aβ_40_ (0–100 equiv; black to pink) in phosphate buffer (5 mM, pH 7.4) at 23 °C. The insert shows changes to the hemin Soret band.

The interaction between hemin (5 μM), which is present as a mixture of dimeric and monomeric species in aqueous media [31,32], and Aβ_40_ was studied at physiologically relevant pH 7.4 in low ionic strength (5 mM) phosphate buffer at 23 °C. Upon adding up to two equivalents of Aβ_40_, the hemin Soret band (*λ*_max_ = 390 nm) was observed to undergo a decrease in molar absorptivity along with a bathochromic shift in the maximum to *λ*_max_ = 394 nm (Figure 1).

These changes in the optical signature are ascribed to the formation of an intermediate species recognized as containing a high-spin, five coordinate 1:1 Aβ_40_: hemin species [13]. In fact, Aβ_40_ can coordinate both the monomeric and dimeric forms of hemin giving two species that are not spectrophotometrically distinguishable (Supplementary Materials, Scheme S1). As the concentrations of Aβ_40_ are raised, a progressive increase in the molar absorptivity is seen in the UV-vis spectrum with an attendant bathochromic shift to *λ*_max_ = 414 nm and the appearance of peaks at *λ* = 532 and 560 nm also being seen. At such concentrations, the low-spin, six coordinated 2:1 Aβ_40_: hemin species predominates [13,18]. The calculated binding constant for the 2:1 Aβ_40_: hemin complex is log β_2_ = 10.84 ± 0.02 (Table 1). By increasing the ionic strength (50 mM phosphate buffer), again at pH 7.4 and 23 °C, similar spectral features were observed (Supplementary Materials, Figure S1). Under these conditions, the calculated binding constant for the 1:1 complex was higher (log *K*_1_ = 7.30 ± 0.02) while that of the 2:1 complex was lower, log β_2_ = 9.77 ± 0.04 (Δlog β_2_ = 1.07). 

Neuroinflammation with attendant acidosis is an established biomarker in neurodegenerative disease [26,27]. Titrations of Aβ_40_ at 23 °C in pH 6.89 and 6.3 phosphate buffer (5 mM) resulted in decreased binding constants of log β_2_ = 9.80 ± 0.01 and 9.05 ± 0.01, respectively (Supplementary Materials, Figure S2). Notably, a significant increase in the formation of the 1:1 Aβ_40_: hemin complex, Δlog *K*_1_ = 1.22, and 2:1 Aβ_40_: hemin complex, Δlog β_2_ = 0.48, was seen upon increasing the temperature of the standard conditions (pH 7.4, 5 mM phosphate buffer) to 37 °C (K_1_ = 7.30 ± 0.02, log β_2_ = 11.32 ± 0.16; Supplementary Materials, Figure S3). In relation to other axial ligands, the log β_2_ value for 50 mM phosphate buffer (pH 7.4) at ambient temperature is still larger than Aβ_16_ (8.82 ± 0.02) [13], L-histidine (2.95) and other histidine-containing peptides (3.91 to 7.67) [33], or the tau repeat peptide I (log *K*_1_ = 4.96 ± 0.01 [34]; log β_2_ could not be determined due to small log *K*_2_ values) studied under comparable conditions. This data is also in agreement with previously reported values for Aβ_40/42_ [14,15,16,17,18,35].

The dynamic equilibrium between low-spin, six coordinate hemin(Aβ_40_)_2_ (1:2) and high-spin, five coordinate hemin-Aβ_40_ (1:1) was also probed qualitatively by decreasing the temperature from 37 to 5 °C at 5, 10, 20, and 50 equiv. of Aβ_40_ (Supplementary Materials, Figure S4). Analogous to the previous work on Aβ_16_-hemin complexes at various temperatures by our group, upon decreasing the temperature, the equilibrium increasingly favours the 2:1 Aβ_40_: hemin complex [13]. At 5 and 10 equiv., upon decreasing the temperature from 37 °C to 4 °C, reversion from the 2:1 Aβ_40_: hemin to a mixed 1:1 and 2:1 Aβ_40_: hemin complex was observed. However, at 20 and 50 equiv., the equilibrium still favored the 2:1 Aβ_40_: hemin complex with a minor 1:1 Aβ_40_: hemin component, as inferred from the observation of the *λ*_max_ at 414 nm signal with a reduced absorption intensity. The partial reversibility of this equilibrium was also demonstrated in the presence of 5 and 10 equivalents of Aβ_40_ by increasing the temperature to 37 °C (ESI Figure S4).

**Figure 2:**
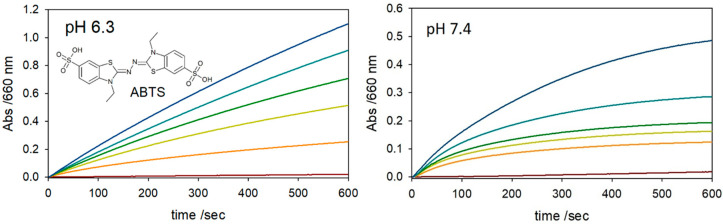
Kinetic profile of ABTS (3 mM) oxidation (*λ* = 660 nm; *ε* = 14700 M^−1^ cm^−1^) [36] with time (0 to 600 s) in phosphate buffer (50 mM, 37 °C) at (**left**) pH 6.3 and (**right**) pH 7.4 as studied in the presence of 2.5 mM H_2_O_2_ (brown) and 2 μM hemin (orange) upon the addition of 2 μM (light green), 10 μM (green), 40 μM (light blue), and 200 μM (blue) Aβ_40_.

The peroxidase activity of Aβ_40_-hemin constructs typically reflect the coordination environment [13,14,15,16,17,18,19,20,21,22,23,24]. We thus sought to investigate how extending the pH and Aβ_40_ concentration ranges, up to 100 equivalents, affected peroxidase activity (Table 2). With such an objective in mind, the peroxidase activity of hemin was measured by monitoring the oxidation of ABTS by hydrogen peroxide at increasing ratios of Aβ_40_: hemin at pH 7.4 in phosphate buffer (50 mM) at 37 °C (Figure 2). The reaction rates were obtained by dividing the slope of the trace in the initial seconds of the reaction by the product of hemin concentration multiplied by the molar extinction coefficient of the ABTS radical cation multiplied by a factor of two to account for the two ABTS radical cations produced during each reaction cycle (cf. Materials and Methods). In this regime, pseudo-first order rate behavior was observed. It was concluded that at higher Aβ_40_ concentrations, where the six-coordinate Aβ_40_: hemin (2:1) species is reported as the predominant species [13], a greater than 2.5- and 3-fold increase in rate was observed at pH 7.4 and 6.3, respectively. 

As can be seen from an inspection of Table 2 (first entry), the catalytic activity of free hemin for the oxidation of ABTS was higher at pH 6.3 (*k*_obs_ = 0.016 s^−1^) than at 7.4 (*k*_obs_ = 0.013 s^−1^). The addition of Aβ_40_ to hemin further enhanced the peroxidase activity at both pH values, with *k*_obs_ always larger at pH 6.3 as compared to 7.4 (Figure 2). Previous studies detailing the oxidative reactivity of Aβ_40_-hemin complexes have failed to explore high concentrations of Aβ_40_ (i.e., more than 20 equivalents) [24]. 

Similar to the trend observed with Aβ_16_ by Flemmig and co-workers [21], in comparison to free hemin, at 100 equiv. of Aβ_40_, the initial rate increased by 2.5- and 3-times at pH 7.4 (0.032 s^−1^) and 6.3 (0.049 s^−1^), respectively. Notably, over the course of the reaction, the rate of peroxidase activity decreased, this is attributed to hemin degradation. The two conditions also showed different rates for decomposition with the larger drop in rate at pH 7.4. At pH 6.3, the peroxidase activity remained high even at longer reaction times, allowing for increased product formation (Figure 2).

Previous work by Ghosh, Dey, and co-workers has shown that Au electrode immobilized hemin-Aβ aggregates can produce partially reduced oxygen species (PROS) [37]. We thus sought to further elaborate on the peroxidase activity of aggregated Aβ-hemin at different pH. Qualitative reactivity experiments utilizing ABTS with hemin and a suspension of mixed soluble and insoluble aggregated Aβ_40_ revealed a rate enhancement versus hemin alone (Supplementary Materials, Figure S5). A faster rate was also again seen at pH 6.3 versus pH 7.4 and when higher levels of Aβ_40_ were present (Supplementary Materials, Figure S6). In order to explore the nature of this activity, hemin-uptake experiments, as measured by following the hemin Soret-band (*λ*_max_ = 390 nm), revealed that at pH 6.3, less hemin was absorbed within the aggregate and at a slower rate (Supplementary Materials, Figure S7). Based on these spectroscopic studies, it was inferred that at pH 6.3 some hemin also leached back into solution after the initial inclusion event. 

**Figure 3:**
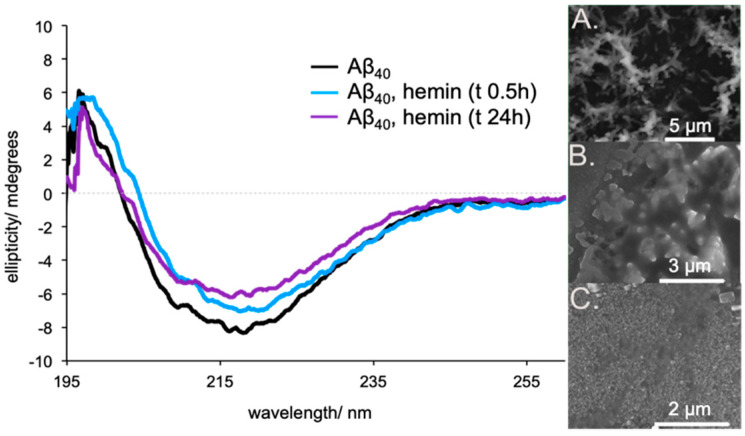
Time-dependent circular dichroism (left) of Aβ_40_ aggregates (black) and after addition of hemin at 0.5 h (blue) and 24 h (purple). SEM analysis (right) of the same (**A**) Aβ_40_ (40 μM) aggregates at pH 7.4 in PBS, (**B**) Aβ_40_ (40 μM) aggregates incubated with hemin (5 μM) for 24 h, and (**C**) freshly dissolved Aβ_40_.

In line with previous work by the groups of Atamna [14] and Xu [35], studies involving the use of circular dichroism (CD) spectroscopy and scanning electron microscopy (SEM) provided support for the inference that hemin can disrupt the Aβ_40_ aggregate architecture (Figure 3). Briefly, upon addition of hemin (5 μM) to Aβ_40_ aggregates (40 μM, 8 equivalents, aged 30 days), the negative CD minimum underwent a slight decrease in intensity with the *λ*_max_ shifting from 218 nm to 220 nm. Over the course of 24 h, a further reduction in the CD intensity was seen with a shift in the minimum to ca. 217 nm.

SEM analyses of the aggregated Aβ_40_ (aged 30 days plus 24 h) sample showed fibrillar constructs, observable at 5 μm. The Aβ_40_ sample (aged 30 days) incubated with hemin (5 μM) for 24 h displayed a more globular like structure, observable at 3 μm. A control experiment involving freshly dissolved Aβ_40_ showed amorphous species, observable at 2 μm. We thus propose that the reactivity of aggregated Aβ_40_-hemin complexes may operate under a mechanism wherein both hemin exposed on the aggregate surface and newly formed soluble oligomeric Aβ_40_-hemin complexes contribute to the peroxidase activity. The ability of acidic pH to disrupt the β sheet architecture is also expected to enhance formation of soluble oligomeric Aβ_40_-hemin complexes with increased peroxidase activity [37,38,39].

The monoamine neurotransmitter, dopamine (DA), plays a key role in proper neuronal function [40]. Recent studies have also demonstrated that DA and oligomeric melanin help maintain standard physiological functions and have a pathological relevance in neurodegenerative conditions [41]. We thus sought to determine how the enhanced peroxidase activity of Aβ_40_-hemin complexes might oxidatively modify DA. The DA modifying activity of these Aβ_40_-hemin complexes was evaluated by monitoring the formation of dopaminochrome, an intermediate during DA oxidation to melanin [42]. Notably, DA oxidation mediated by hemin and Aβ_40_-hemin complexes did not exhibit a simple Michaelis-Menten behavior, but instead showed evidence of a substrate inhibition effect (ESI Figure S8). The kinetic constants (pseudo-first order approach) (Table 3) were obtained by dividing the slope of the trace in the initial seconds of the reaction by the product of hemin concentration multiplied by the molar extinction coefficient of dopamine (cf. Materials and Methods).

**Figure 4:**
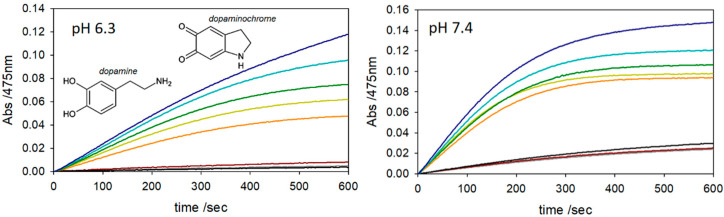
Kinetic profile of DA (3 mM) oxidation (*λ* = 470 nm; *ε* = 3300 M^−1^ cm^−1^) [43] with time (0 to 600 s) in phosphate buffer (50 mM, 37 °C) at (**left**) pH 6.3 and (**right**) pH 7.4 in the presence of hemin (2 μM) (brown trace) or hydrogen peroxide (2.5 mM, black). Traces were recorded after the addition of both hemin and hydrogen peroxide (orange) and upon the addition of 2 µM (light green), 10 µM (green), 40 µM (light blue) and 200 µM Aβ_40_ (blue). The autoxidation trace is shown as a grey line.

At pH 7.4 in phosphate buffer (50 mM), Aβ_40_ increases the peroxidase activity of hemin in terms of DA oxidation, albeit the rate at 100 equivalents of Aβ_40_ is only 1.5-times greater than hemin alone (Figure 4). At pH 6.3, DA oxidation becomes more difficult due to the proton-dependent increase in the substrate E° value [44]. This difference is reflected in the hemin-mediated oxidation of DA that proceeds at a 3-times slower rate of 0.018 s^−1^ at pH 6.3 vs. 0.053 s^−1^ at pH 7.4 (Figure 4). However, at pH 6.3, the presence of Aβ_40_ (100 equivalents) increased the rate almost two-fold.

Similar studies utilizing the Aβ_16_ model system showed marked differences versus Aβ_40_. At both pH 7.4 and 6.3, Aβ_16_ proved more effective at increasing the rate of hemin-catalyzed oxidation of ABTS and DA in the presence of H_2_O_2_ (Table 2 and Table 3, Figures S9–S12). In particular, Aβ_16_ showed a significant rate enhancement with a 4-fold and almost 6-fold increase of ABTS oxidation (hemin vs. 100 equiv. Aβ_16_) at pH 7.4 and 6.3, respectively. In the case of DA oxidation, Aβ_16_ displayed a 3-fold and 4-fold increase at pH 7.4 and 6.3, respectively.

These findings may be rationalized by assuming mechanistically that the peroxidase-like activity of hemin resembles that of the peroxidase enzyme [45]. Briefly, there is an initial activation of H_2_O_2_ with attendant formation of a high valent P^•+^Fe^IV^=O species (Compound I), where P^•+^ indicates the porphyrin *π*-radical cation (Equation (1)). The involvement of compound I-like oxidant for heme-Aβ peptides complexes has been recently characterized [46]. The formation of this active species is followed by two one-electron oxidations of two substrate molecules (SH) (Equations (2) and (3)). The rate-determining step (rds) of the catalytic cycle depends on the relative concentrations of H_2_O_2_ and SH. The experiments reported here were performed under conditions where the H_2_O_2_ concentration has reached kinetic saturation, making step 3 the rds. The data supports the conclusion that the rate increase seen with a large excess of Aβ_40_ is due to faster electron transfer from SH to the Fe^IV^=O species (Equation (3)).

PFe^III^ + H_2_O_2_ ⇄ [PFe^III^-H_2_O_2_] → P^•+^Fe^IV^ = O + H_2_O (1)

P^•+^Fe^IV^ = O +SH → PFe^IV^ = O + S^•^ + H^+^ (2)

PFe^IV^ = O + SH + H^+^ → PFe^III^ + S^•^ + H_2_O (3)

The effect of Aβ peptides in promoting a marked rate increase may be ascribed to the following three hypotheses: (i) axial coordination of the Fe^III^ center to enhance oxidative reactivity, (ii) acid/ base catalysis facilitating the release of the oxo group as water, and (iii) enhancing substrate approach to the iron-oxo porphyrin. In order to assess the relative contributions of the previous hypotheses, analogous DA oxidation experiments were carried out using hemin-glycyl-L-histidine methyl ester (hemin-GH) [47]. The appended histidine residue is strongly bound to the iron(III) centre via an intramolecular coordination, leading to a five-coordinate iron(III) species with enhanced peroxidase activity. Not surprisingly, the rate of DA oxidation was more than one order of magnitude larger (0.785 s^−1^) than that of free hemin under the same conditions (0.053 s^−1^) (Supplementary Materials, Table S1). The addition of 1 or 5 equivalents Aβ_16_ failed to modify the reaction rate significantly, with values of 0.819 s^−1^ and 0.824 s^−1^, respectively, being observed (Supplementary Materials, Figure S13). However, addition of 20 and 100 equivalents Aβ_16_ caused a rate decrease, with values of 0.773 s^−1^ and 0.591 s^−1^, respectively, now being obtained (Table S1). This leads us to suggest that the unstructured organization of the Aβ_16_ peptide chain around the hemin centre (hypotheses ii and iii) has little effect, if any, on the reaction rates. High levels of peptide are believed to introduce some steric hindrance, but the principal effect leading to the increase in activity is ascribed by default to iron chelation by the Aβ peptide (hypothesis i).

To the extent such considerations are correct, the mechanism of substrate oxidation by hemin in the presence of Aβ peptides must include a pre-equilibrium step leading to a mixture of hemin, hemin-Aβ, and hemin(Aβ)_2_, defined by binding constants *K*_1_ and *K*_2_ (Supplementary Materials, Scheme S2). Upon reacting with H_2_O_2_, hemin is transformed into the iron-oxo species through pathway a. Hemin-Aβ and hemin(Aβ)_2_ are transformed into the same active iron-oxo species through pathways b and c. The reversible disassociation of six-coordinated hemin(Aβ)_2_ in the pre-equilibrium prevents reaction inhibition and ultimately yields the same active intermediate formed by hemin-Aβ (1:1). At saturating levels of H_2_O_2_, the distal histidine of the second Aβ peptide is also readily displaced by H_2_O_2_. The first Aβ peptide has been shown to coordinate the iron center strongly via one of the three histidine residues (preferably His13) [18]. This, in turn, increases the electron density at iron and favours electron transfer from the substrate to the iron-oxo species (hypothesis i).

A comparison between the two amyloid peptide species considered in the present study reveals that hemin has a higher affinity (*K*_1_) for Aβ_40_ over Aβ_16_ (Table 1). However, analysis of the *k*_obs_ values reported in Table 2 and Table 3 indicate a higher activating effect (peroxidase activity) in the case of Aβ_16_. This phenomenon is attributed to increased steric bulk around the metal center caused by the larger Aβ_40_ peptide, something that prevents substrate approach and electron transfer. It should also be noted that a larger concentration of the Aβ is required for complete binding to hemin under conditions of catalysis than might be expected based on the value for log *K*_1_. This is rationalized in terms of a high substrate concentration that competitively prevents binding the second hemin axial site. This, in turn, precludes catalyst activation with H_2_O_2_ and is also reflected in the inhibitory effect of excess DA.

Finally, we evaluated the competitive self-oxidation of Aβ in the presence of DA and H_2_O_2_. Previous work by our group [13,48], as well as others [49], has demonstrated that the Aβ peptide is a substrate for metal-catalyzed oxidation. Briefly, a reaction mixture of hemin (6 μM), Aβ_16_ (30 μM), H_2_O_2_ (1 mM) and DA (3 mM) in phosphate buffer (50 mM, pH 7.4) was incubated at 37 °C for 30 and 120 min, followed by HPLC-MS analysis. As shown in Table S2 and Figure S14, the peptide remains mostly unmodified (79% intact) at both 30 and 120 min. Control experiments without DA showed that the modification pattern is similar, 85% unmodified Aβ_16_, indicating that the oxidation is mainly mediated by the oxidative hemin-Aβ_16_-H_2_O_2_ system (Table S3). The similar amounts of modified peptide seen for the two reaction times leads us to suggest rapid modification of Aβ_16_ followed by catalyst decomposition or inhibition in the case of added DA.

## 3. Materials and Methods

### 3.1. General Procedures

Hemin, 2,2′-azino-bis(3-ethylbenzothiazoline-6-sulphonic acid) (ABTS), and dopamine hydrochloride were purchased from Sigma Aldrich and used as received. Hemin stock solutions were prepared by dissolving hemin in 0.5 M NaOH in Milli Q water and centrifuging at 14,000 rpm for 2 min. The supernatant was decanted and diluted in Milli Q water to yield an approximately 3 mM stock solution. The phosphate buffer solution was prepared by dissolving the appropriate amounts of Na_2_HPO_4_ and NaH_2_PO_4_ then adjusting the pH with concentrated NaOH (aq.). Aβ_16_ was prepared as previously reported [48]. Aβ_40_ was prepared via Fmoc amino solid-phase peptide synthesis using a Liberty Blue™ microwave peptide synthesizer [50]. Preparative RP-HPLC purification of peptides was performed using an Agilent Zorbax SB-C_18_ Prep HT column 21.2 × 250 mm. Analytical RP-HPLC characterization of peptides was performed using an Agilent Zorbax column 4.6 × 250 mm. An Agilent Technologies 6530 Accurate Mass QT of LC/MS was used for high-resolution mass spectra of purified peptides. Solvents used were HPLC grade. Aβ_40_ stock solutions for UV-vis titrations were prepared by dissolving the peptide in Milli Q water. The solution was sonicated and vortex mixed until the peptide was dissolved. This mixture was filtered through a 0.22 micron PTFE filter. Concentration of the Aβ_40_ peptide was measured by UV-vis spectroscopy using a molar extinction coefficient ε_280_ = 1480 M^−1^ cm^−1^ [51]. Concentration of hemin solution was measured by UV-vis spectroscopy using a molar extinction coefficient ε_390_ = 66,000 M^−1^ cm^−1^ [52]. UV-vis spectra and kinetic experiments were recorded on a Hewlett Packard HP 8453A diode array spectrophotometer equipped with a thermostated, magnetically stirred cuvette holder. The peptides were studied by direct LC-MS/MS analysis. LC-MS and LC-MS/MS data were obtained by using an LCQ DECA ion-trap mass spectrometer with an ESI ion source, coupled with an automatic injector Surveyor HPLC system and controlled by the Xcalibur 1.3 software (Thermo-Finnigan, San Jose, CA, USA). The system was run in an automated LC-MS/MS mode and by using a Surveyor HPLC system (Thermo-Finnigan, San Jose, CA, USA) equipped with a BIOBASIC C18 column (5 µM), 150 × 2.1 mm). For analysis of oxidation studies, the acquired MS/MS spectra were automatically searched against the human Aβ_16_ sequence by using the SEQUEST algorithm to identify the modified residues. This algorithm was incorporated into the Bioworks 3.1 software (Thermo-Finnigan, San Jose, CA, USA). SEM analyses were prepared by drop-casting samples onto silica wafers (University Wafers) then gently drying with nitrogen. Images were taken on a FEI Quanta 650 ESEM instrument in The University of Texas at Austin–Texas Materials Institute. Circular dichroism (CD) analyses were carried out using a Jasco J-815 CD spectrometer acquired through The University of Texas at Austin Texas Institute for Drug Discovery & Development (Ti3D). Titration data were processed with the Hyperquad package [53] using a range of wavelengths centered around the two absorption maxima. The fittings allowed the determination of the equilibrium constants for the two-step binding processes:
*Step 1:*   [hemin(H_2_O)_2_] + Aβ_40_ ⇄ [hemin(Aβ_40_)(H_2_O)] + H_2_O  (*K*_1_)*Step 2:*   [hemin(Aβ_40_)(H_2_O)] + Aβ_40_ ⇄ [hemin(Aβ_40_)_2_] + H_2_O  (*K*_2_)*Global:*   [hemin(H_2_O)_2_] + 2 Aβ_40_ ⇄ [hemin(Aβ_40_)_2_] + 2 H_2_O  (β_2_) 

### 3.2. Kinetics of ABTS Oxidation

The catalytic oxidation of ABTS by a hemin-hydrogen peroxide mixture was studied at 37 °C in 50 mM phosphate solution at pH 7.4 or 6.3. ABTS (3 mm) oxidation was initiated by the addition of hemin (3 µM), H_2_O_2_ (2.5 mM) and amyloid-β fragments 1–16 and 1–40 (0–200 µM). Each mixture containing hemin and buffer solution was equilibrated at 37 °C for 5 min before the addition of the substrate and peptide; hydrogen peroxide was added to the reaction solution as the last reagent. The reactions were followed through the development of the optical band of ABTS^+·^ radical cation at 660 nm (ε_660_ = 14,700 M^−1^ cm^−1^) [36]. Reaction rates in Δ(absorbance/s) units were calculated from the slope of the trace in the initial 30 s, with the first few seconds discarded to allow stabilization of the readings, and were then converted into the observed kinetic constant (s^−1^) by dividing the calculated value over the product of catalyst concentration multiplied by the molar extinction coefficient of ABTS^·+^ multiplied by a factor of two (each catalytic cycle involves formation of two ABTS^·+^ molecules).

### 3.3. Kinetics of Dopamine (DA) Oxidation

The catalytic oxidation of DA by a hemin-hydrogen peroxide mixture was studied at 37 °C in 50 mM phosphate solution at pH 7.4 or 6.3. DA (3 mm) oxidation was initiated by the addition of hemin (3 µM), H_2_O_2_ (2.5 mM) and amyloid-β fragments 1–16 and 1–40 (0–200 µM). Each mixture containing hemin and buffer solution was equilibrated at 37 °C for 5 min before the addition of the substrate and peptide; hydrogen peroxide was added to the reaction solution as the last reagent. The reactions were followed via the development of the optical band of dopaminochrome at 470 nm (ε_470_ = 3300 M^−1^ cm^−1^) [43]. Reaction rates in Δ(absorbance/s) units were calculated from the slope of the trace in the initial 30 s, with the first few seconds discarded to allow stabilization of the readings, and were then converted into the observed kinetic constant (s^−1^) by dividing by the product of the catalyst concentration multiplied by the molar extinction coefficient of dopaminochrome.

### 3.4. Aggregate Formation

The aggregated form of Aβ_40_ was prepared as follows: a stock solution of Aβ_40_ was prepared by dissolving Aβ_40_ in DMSO (ca. 700 µM). The mixture was sonicated and vortex mixed until the peptide was solubilized. This solution was diluted with 20 mM phosphate buffer solution (pH 7.5), to give a final concentration of <2% DMSO, and incubated at 37 °C for 7 days. The formation of insoluble aggregate species was observed, as seen by the appearance of insoluble Aβ_40_ species. The mixed soluble and insoluble Aβ_40_ aggregate solution was split into Eppendorf tubes and centrifuged down. The supernatant was decanted and the solid was washed three times with Milli Q water (1 mL) to remove any salt and DMSO. The aggregate was resuspended in a UV-vis vial and utilized for the peroxidase activity studies. Two sample concentrations were prepared by varying the level of dilution.

### 3.5. Aβ_16_ Modification in the presence of Hydrogen Peroxide and Hemin by LC-MS

Competitive peptide modification was studied using HPLC-ESI/MS. Samples were prepared by mixing hemin (6 μM), H_2_O_2_ (1 mM), Aβ_16_ (30 μM), and DA (3 mM) in phosphate buffer solution (50 mM) pH 7.4 and incubating at 37 °C. HPLC-ESI/MS analysis were performed at different reaction times. LC-MS data were obtained by using a LCQ ADV MAX ion-trap mass spectrometer. The elution of Aβ_16_ was carried out by using 0.1% HCOOH in distilled water (solvent A) and 0.1% HCOOH in acetonitrile (solvent B), with a flow rate of 0.2 mL/ min. The solvent gradient started with 98% solvent A for 5 min followed by a linear gradient from 98 to 55% A over 65 min.

## 4. Conclusions

In conclusion, we have demonstrated that the amyloid peptide Aβ_40_ displays an environmental dependence on the dynamic equilibrium between high-spin five-coordinate hemin-Aβ_40_ and low-spin six-coordinate hemin(Aβ_40_)_2_ species and that this affects the peroxidase activity of these species. Hemin was also shown to disrupt Aβ_40_ aggregated structures and display enhanced peroxidase activity in the presence of Aβ_40_ aggregates versus hemin alone. This work thus sets the stage for understanding in greater detail the role of aberrant metal-ions (e.g., Fe, Cu, and Zn) in relationship to both full length and smaller hydrolyzed Aβ peptides found in Alzheimer’s disease brains and the role of the associated complexes in enhancing reactive oxygen (ROS) and nitrogen species (RNS) that are considered causal in CNS neurodegeneration [54]. Furthermore, within the context of this and related studies, the enhanced peroxidase activity of hemin-peptide constructs are counterproductive to normal healthy activity, wherein cells maintain an array of corrective mechanisms to prevent cellular and organismal death. New studies designed to understand the specific processes resulting in these phenomena (i.e., the release of amyloid beta peptides and dysregulation of hemin pathways resulting in hemin-amyloid beta species) may uncover key processes related to the origins of Alzheimer’s and related neurodegenerative diseases.

## Figures and Tables

**Table 1:** Equilibrium constants for hemin binding to Aβ_40_.

Complex ^a^	log *K*_1_	log *K*_2_^b^	log β_2_
[hemin(Aβ_40_)_2_]	6.08 ± 0.02	4.77	10.84 ± 0.02
[hemin(Aβ_40_)_2_] ^c^	5.60 ± 0.01	4.20	9.80 ± 0.01
[hemin(Aβ_40_)_2_] ^d^	4.76 ± 0.03	4.30	9.05 ± 0.01
[hemin(Aβ_40_)_2_] ^e^	5.30 ± 0.02	4.47	9.77 ± 0.04
[hemin(Aβ_40_)_2_] ^f^	7.30 ± 0.02	4.02	11.32 ± 0.16
[hemin(Aβ_16_)_2_] ^g^	4.80 ± 0.02	4.03	8.82 ± 0.02

^a^ Standard conditions: hemin (5 μM) in pH 7.4 phosphate buffer (5 mM) at 23 °C. ^b^ Obtained as the difference between log β_2_ and log *K*_1_. ^c^ pH 6.89. ^d^ pH 6.3. ^e^ 50 mM phosphate buffer pH 7.4. ^f^ 37 °C. ^g^ 50 mM PBS, pH 7.5, 27 °C; [13].

**Table 2:** Kinetic constants for the oxidation of ABTS (*λ* = 660 nm; *ε* = 14,700 M^−1^ cm^−1^) [36] by H_2_O_2_ (2.5 mM) and hemin (2 μM) at 37 °C in the presence of varying amounts of Aβ_40_ or Aβ_16_ at pH 7.4 or 6.3. The calculated variability based on 2 independent measurements was equal to or less than 0.001 s^−1^.

Species	pH 7.4−Aβ_16_ *k*_obs_ (s^−1^)	pH 7.4−Aβ_40_ *k*_obs_ (s^−1^)	pH 6.3−Aβ_16_ *k*_obs_ (s^−1^)	pH 6.3−Aβ_40_ *k*_obs_ (s^−1^)
Hemin	0.013	0.013	0.016	0.016
Aβ (1 equiv.)	0.022	0.018	0.034	0.027
Aβ (5 equiv.)	0.022	0.021	0.042	0.033
Aβ (20 equiv.)	0.032	0.027	0.053	0.038
Aβ (100 equiv.)	0.054	0.032	0.092	0.049

**Table 3:** Kinetic constants for the oxidation of DA (3 mM, *λ* = 470 nm; *ε* = 3300 M^−1^ cm^−1^) [43] by H_2_O_2_ (2.5 mM) and hemin (2 μM) at 37 °C with varying amounts of Aβ_40_ or Aβ_16_ in phosphate buffer (50 mM) at pH 7.4 or 6.3. The calculated variability, based on at least 2 independent measurements, was in all cases ≤ 0.001 s^−1^.

Species	pH 7.4−Aβ_16_ *k*_obs_ (s^−1^)	pH 7.4−Aβ_40_ *k*_obs_ (s^−1^)	pH 6.3−Aβ_16_ *k*_obs_ (s^−1^)	pH 6.3−Aβ_40_ *k*_obs_ (s^−1^)
Hemin	0.053	0.052	0.018	0.018
Aβ (1 equiv.)	0.049	0.058	0.024	0.023
Aβ (5 equiv.)	0.056	0.060	0.023	0.027
Aβ (20 equiv.)	0.077	0.068	0.037	0.031
Aβ (100 equiv.)	0.171	0.076	0.076	0.034

## Supplementary Materials

The following are available online. Figure S1. Hemin-Ab40 spectroscopic titrations carried out in 5 mM phosphate buffer solution (left) and 50 mM phosphate buffer solution (right), Figure S2. Hemin-Ab40 spectroscopic titrations carried out at pH 6.3 (left) and 6.89 (right) phosphate buffer solution (5 mM), Figure S3. Hemin-Ab40 spectroscopic titrations carried out at (left) room temperature and (right) 37 °C in 5 mM phosphate buffer solution, Figure S4. Temperature dependent reversibility of the hemin-Ab40 complex formation as inferred from UV-vis spectroscopic studies in 5 mM phosphate buffer solution, Figure S5. Reactivity (ABTS oxidation) of the Ab40 aggregate at pH 6.3 (red) versus pH 7.4 (grey) phosphate buffer solution (50 mM) as determined by monitoring the absorption intensity at 660 nm as a function of time, Figure S6. Reactivity (ABTS oxidation) of the Ab40 aggregate (red) versus diluted aggregate (grey) and hemin (black) as determined by monitoring the absorption intensity at 660 nm as a function of time in pH 7.4 phosphate buffer solution (50 mM), Figure S7. (top) Hemin uptake within Ab40 aggregates at pH 6.3 (red) and 7.4 (grey) phosphate buffer solution (50 mM) at 660 nm and (bottom) UV-vis traces following the change absorption within the Ab40 aggregate as a function of time starting at t = 0 (black) and ending at t = 900 s (purple), Figure S8. (Left) Kinetic profile of DA oxidation with time in 50 mM phosphate buffer solution at pH 7.5 and 37 °C in presence of hemin (2 μM), hydrogen peroxide (2.5 mM), Ab16 (10 μM), and varying concentration of DA (0.2 mM, blue trace; 0.5 mM, brown; 1 mM, green; 2 mM, orange and 3 mM, light green) as determined by monitoring the absorption intensity at 475 nm as a function of time. (Right) Dependence of the reaction rate of dopaminochrome formation on the concentration of DA, Figure S9. Kinetic profile of ABTS (3 mM) oxidation with time in 50 mM phosphate buffer solution at pH 7.4 and 37 °C in presence of hydrogen peroxide (2.5 mM) (brown trace) and hemin (2 μM, orange) and upon the addition of 2 μM (light green), 10 μM (green), 40 μM (light blue) and 200 μM Aβ16 (blue) as determined by monitoring the absorption intensity at 660 nm as a function of time, Figure S10. Kinetic profile of ABTS (3 mM) oxidation with time in 50 mM phosphate buffer solution at pH 6.3 and 37 °C in presence of hydrogen peroxide (2.5 mM) (brown trace) and hemin (2 μM, orange) and upon the addition of 2 μM (light green), 10 μM (green), 40 μM (light blue) and 200 μM Aβ16 (blue) as determined by monitoring the absorption intensity at 660 nm as a function of time, Figure S11. Kinetic profile of DA (3 mM) oxidation with time in 50 mM phosphate buffer solution at pH 7.4 and 37 °C in presence of hemin (2 μM) (brown trace) or hydrogen peroxide (2.5 mM, black), after the addition of both hemin and peroxide (orange) and upon the addition of Aβ16 at 2 μM (light green), 10 μM (green), 40 μM (light blue) and 200 μM (blue) as determined by monitoring the absorption intensity at 475 nm as a function of time. The autoxidation trace is shown as grey curve, Figure S12. Kinetic profile of DA (3 mM) oxidation with time in 50 mM phosphate buffer solution at pH 6.3 and 37 °C in presence of hemin (2 μM) (brown trace) or hydrogen peroxide (2.5 mM, black), after the addition of both hemin and peroxide (orange) and upon the addition of Aβ16 2 μM (light green), 10 μM (green), 40 μM (light blue) and 200 μM (blue) as determined by monitoring the absorption intensity at 475 nm as a function of time. The autoxidation trace is shown as grey curve, Figure S13. Kinetic profile of DA (3 mM) oxidation with time in 50 mM phosphate buffer solution at pH 6.3 and 37 °C in presence of hemin-glycyl-L-histidine methyl ester (hemin-GH) (2 μM) (brown trace) or hydrogen peroxide (2.5 mM, black), after the addition of both hemin-GH and hydrogen peroxide (orange) and upon the addition of 2 μM (light green), 10 μM (green), 40 μM (light blue) and 200 μM Aβ16 (blue) as determined by monitoring the absorption intensity at 475 nm as a function of time. The autoxidation trace is shown as a grey curve, Figure S14. HPLC-MS elution profiles of Aβ16 (30 μM) in phosphate buffer solution (50 mM) pH 7.4 in the presence of hemin (6 μM), H2O2 (1 mM) and DA (3 mM) after 30 min (left panel) and 120 min (right panel) reaction time at 37 °C; Table S1. Kinetic constants for the oxidation of DA (l = 470 nm; e = 3300 M-1cm-1)d by hydrogen peroxide and hemin-glycyl-L-histidine methyl ester (hemin-GH) with varying amounts of Ab16 according to the following conditions: hemin-GH (2 μM), DA (3 mM), H2O2 (2.5 mM) in pH 6.3 phosphate buffer solution (50 mM) at 37 °C. The calculated variability for all cases was equal to or less than 0.001 s-1 as based on at least 2 repeat measurements, Table S2. Modification of Aβ16 peptide as inferred from HPLC-MS analysis observed upon reaction of hemin (6 μM), Aβ16 (30 μM), hydrogen peroxide (1 mM) and DA (3 mM) in pH 7.4 phosphate buffer solution (50 mM) at 37 °C, Table S3. Modification of Aβ16 peptide as inferred from HPLC-MS analysis upon reaction of hemin (6 μM), Aβ16 (30 μM) and hydrogen peroxide (1 mM) in phosphate buffer solution (50 mM) pH 7.4 at 37 °C; Scheme S1. Proposed equilibria between hemin, dimeric [hemin]2, dimeric [hemin]2-Aβ40 (2:1), hemin-Aβ40 (1:1), and hemin(Aβ40)2 (1:2), Scheme S2. Proposed reaction mechanism explaining the observed peroxidase-like reactivity of hemin-Aβ40 complexes. K1 and K2 are the equilibrium constants for heme binding to one and two equivalents of Ab40 or Ab16, respectively, and SH is a generic substrate. Path a, path b, and path c indicate the reaction mechanism of H2O2 with free hemin, [hemin(Ab)] complex and [hemin(Ab)2] complex, respectively.
