# Supplementary material for: Condition-Dependent Coordination and Peroxidase Activity of Hemin-Aβ Complexes
Source: Molecules. 2020 Oct 30;25(21):5044. doi: 10.3390/molecules25215044 (PMC7662341; doi:10.3390/molecules25215044)
Supplement: Supplementary file 1 [file molecules-25-05044-s001.pdf]

Condition-dependent coordination and peroxidase activity of hemin-Aβ complexes

Chiara Bacchella,[1,†] James T. Brewster, II,[2,†] Steffen Bähring,[3,*] Simone Dell'Acqua,[1] Harrison D. Root,[2] Gregory D. Thiabaud,[2] James F. Reuther,[2,4] Enrico Monzani,[1] Jonathan L. Sessler[2,*], and Luigi Casella[1,*]

1 Department of Chemistry, University of Pavia, Via Taramelli 12, 27100 Pavia, Italy
2 Department of Chemistry, The University of Texas at Austin, 105 East 24th, Street-Stop A5300, Austin, Texas 78712-1224, United States of America
3 Department of Physics, Chemistry, and Pharmacy, University of Southern Denmark, Campusvej 55, Odense, DK-5230, Denmark
4 Department of Chemistry, University of Massachusetts Lowell, Lowell, MA 01854, United States of America
* Correspondence: sbahring@sdu.dk (S.B.); sessler@cm.utexas.edu (J.L.S.); luigi.casella@unipv.it (L.C.)
† These authors contributed equally.

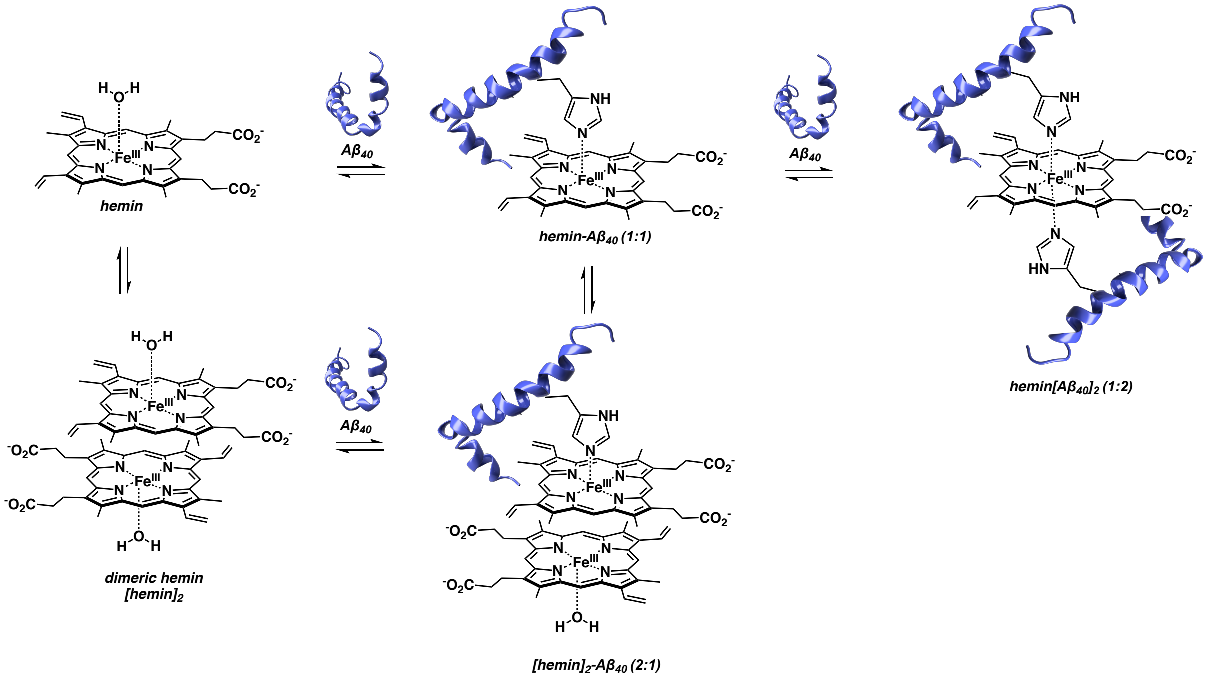

Scheme S1. Proposed equilibria between hemin, dimeric [hemin]$_2$, dimeric [hemin]$_2$-Aβ$_{40}$ (2:1), hemin-Aβ$_{40}$ (1:1), and hemin(Aβ$_{40}$)$_2$ (1:2).

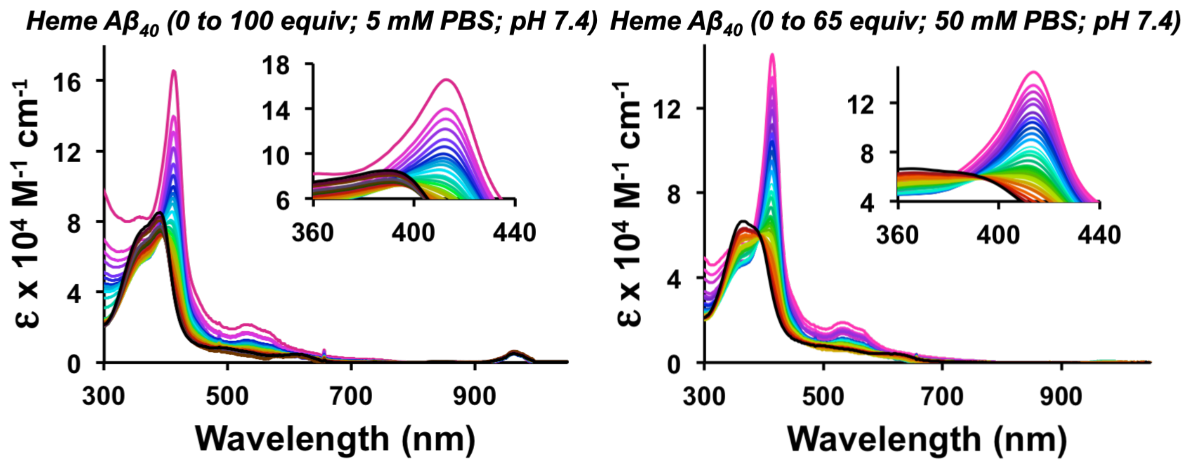

Figure S1. Hemin-Aβ_{40} spectroscopic titrations carried out in 5 mM phosphate buffer solution (left) and 50 mM phosphate buffer solution (right).

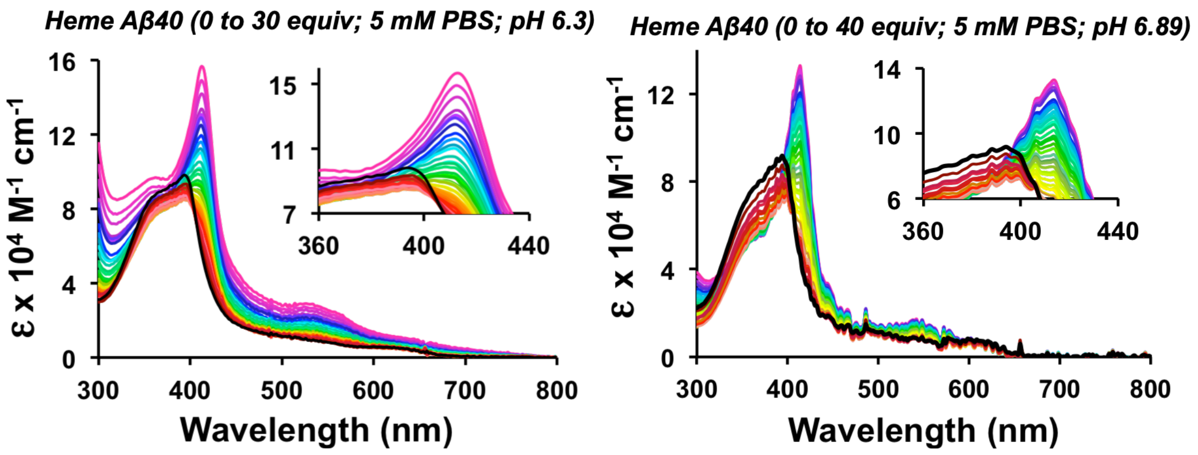

Figure S2. Hemin-Aβ_{40} spectroscopic titrations carried out at pH 6.3 (left) and 6.89 (right) phosphate buffer solution (5 mM).

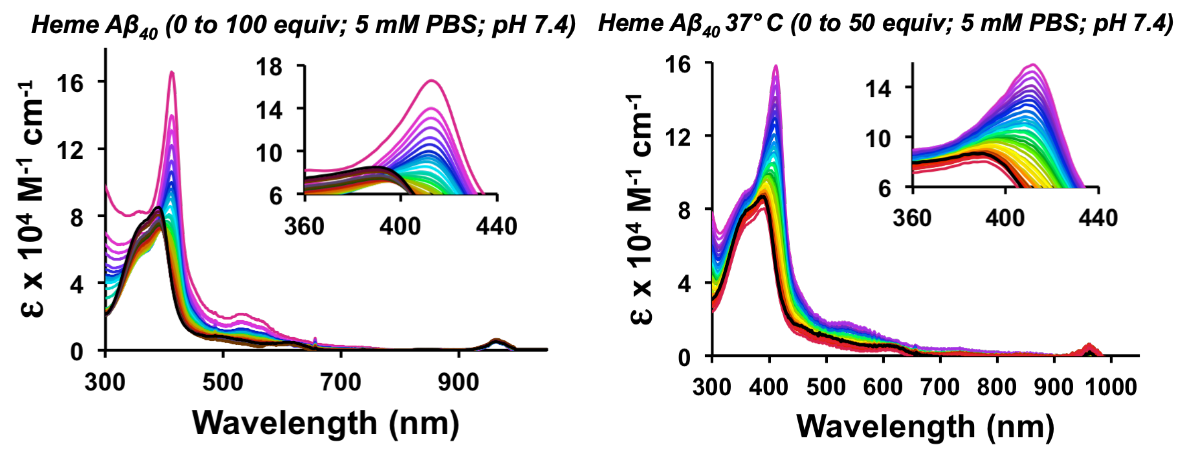

Figure S3. Hemin-Aβ_{40} spectroscopic titrations carried out at (left) room temperature and (right) 37 °C in 5 mM phosphate buffer solution.

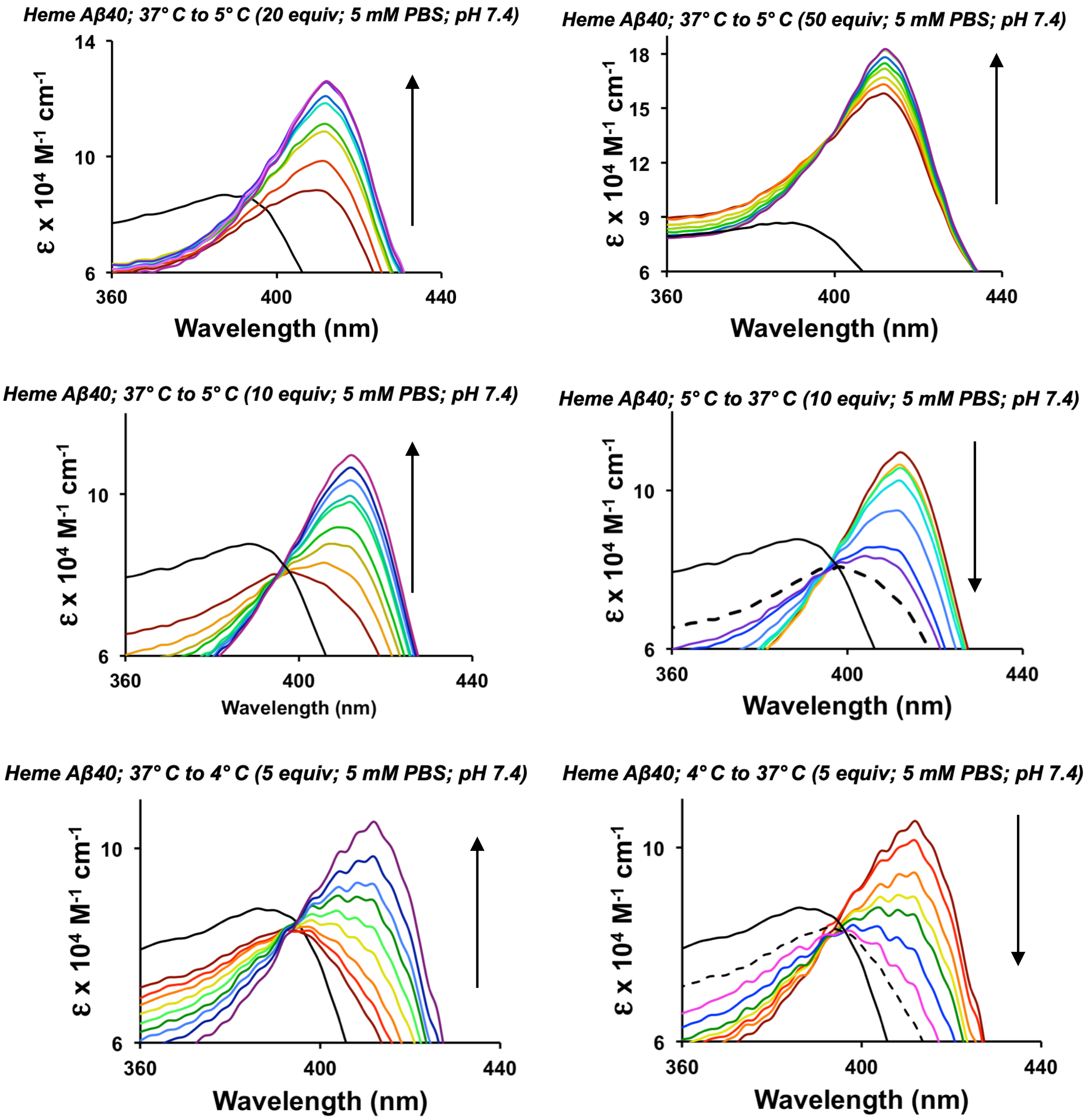

Figure S4. Temperature dependent reversibility of the hemin-Aβ_{40} complex formation as inferred from UV-vis spectroscopic studies in 5 mM phosphate buffer solution.

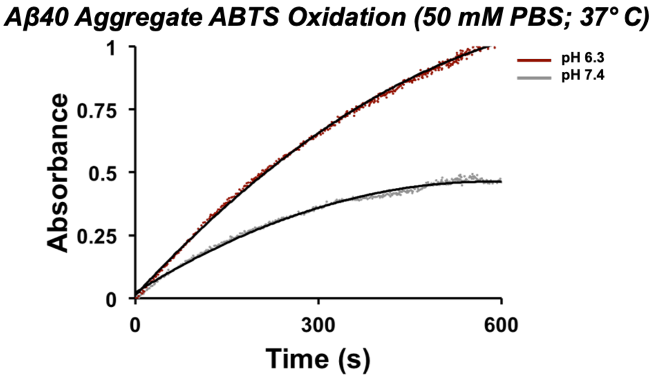

Figure S5. Reactivity (ABTS oxidation) of the Aβ_{40} aggregate at pH 6.3 (red) versus pH 7.4 (grey) phosphate buffer solution (50 mM) as determined by monitoring the absorption intensity at 660 nm as a function of time.

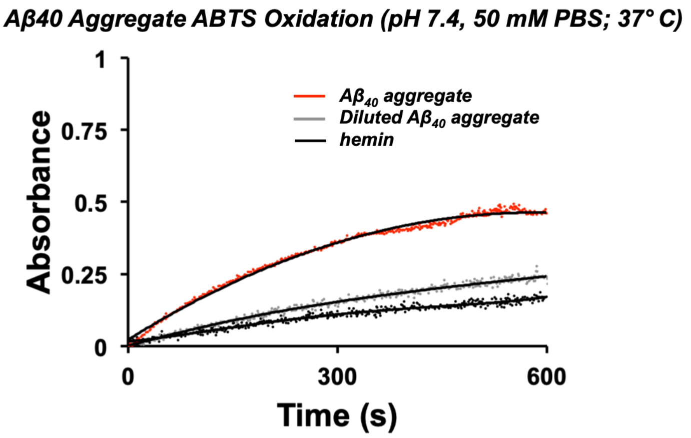

Figure S6. Reactivity (ABTS oxidation) of the Aβ40 aggregate (red) versus diluted aggregate (grey) and hemin (black) as determined by monitoring the absorption intensity at 660 nm as a function of time in pH 7.4 phosphate buffer solution (50 mM).

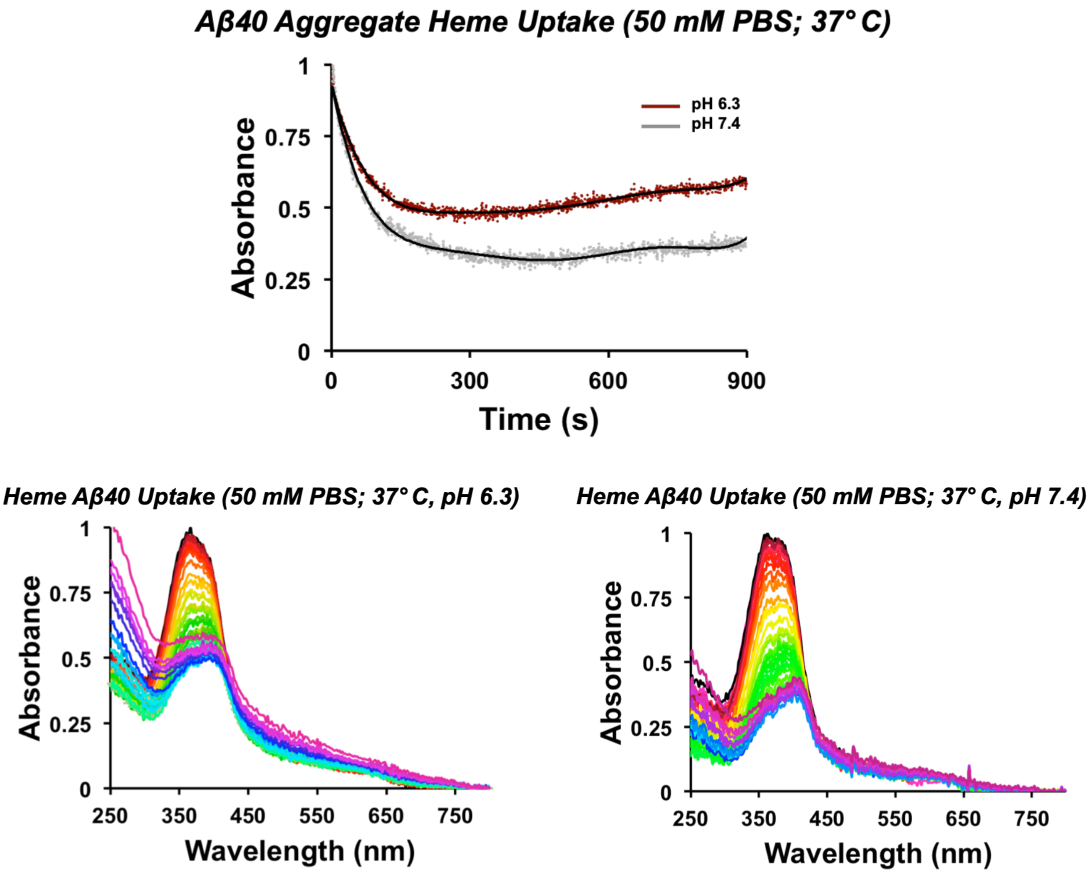

Figure S7. (top) Hemin uptake within Aβ40 aggregates at pH 6.3 (red) and 7.4 (grey) phosphate buffer solution (50 mM) at 660 nm and (bottom) UV-vis traces following the change absorption within the Aβ40 aggregate as a function of time starting at t = 0 (black) and ending at t = 900 s (purple).

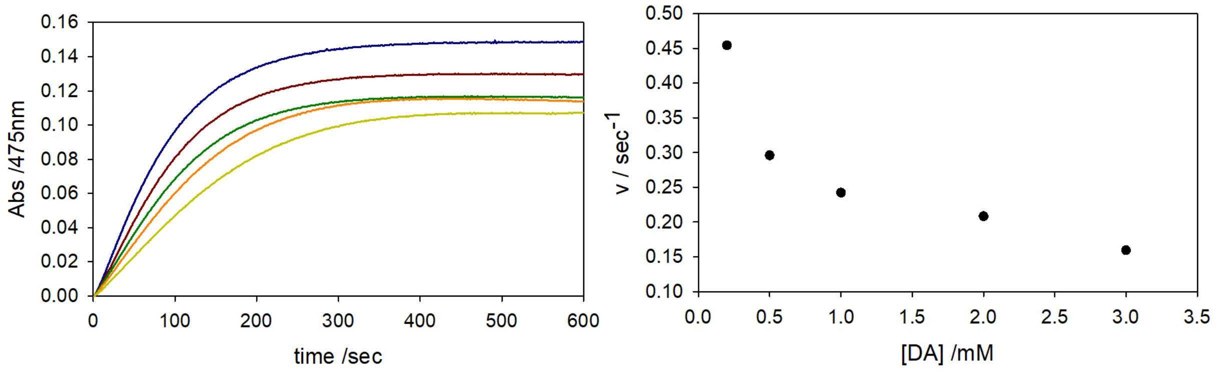

Figure S8. (Left) Kinetic profile of DA oxidation with time in 50 mM phosphate buffer solution at pH 7.5 and 37 °C in presence of hemin (2 μM), hydrogen peroxide (2.5 mM), Aβ_{16} (10 μM), and varying concentration of DA (0.2 mM, blue trace; 0.5 mM, brown; 1 mM, green; 2 mM, orange and 3 mM, light green) as determined by monitoring the absorption intensity at 475 nm as a function of time. (Right) Dependence of the reaction rate of dopaminochrome formation on the concentration of DA.

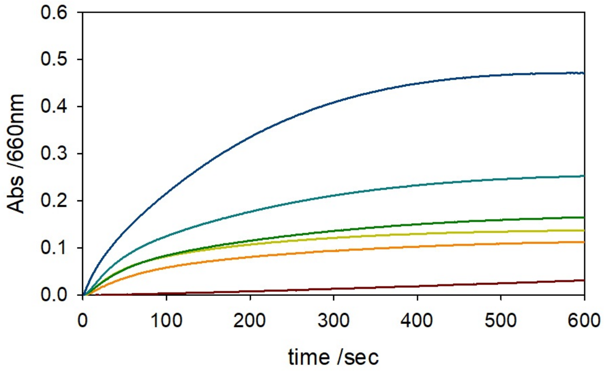

Figure S9. Kinetic profile of ABTS (3 mM) oxidation with time in 50 mM phosphate buffer solution at pH 7.4 and 37 °C in presence of hydrogen peroxide (2.5 mM) (brown trace) and hemin (2 μM, orange) and upon the addition of 2 μM (light green), 10 μM (green), 40 μM (light blue) and 200 μM Aβ_{16} (blue) as determined by monitoring the absorption intensity at 660 nm as a function of time.

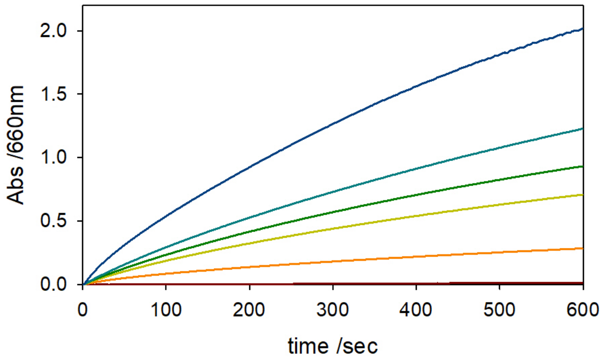

Figure S10. Kinetic profile of ABTS (3 mM) oxidation with time in 50 mM phosphate buffer solution at pH 6.3 and 37 °C in presence of hydrogen peroxide (2.5 mM) (brown trace) and hemin (2 μM, orange) and upon the addition of 2 μM (light green), 10 μM (green), 40 μM (light blue) and 200 μM Aβ_{16} (blue) as determined by monitoring the absorption intensity at 660 nm as a function of time.

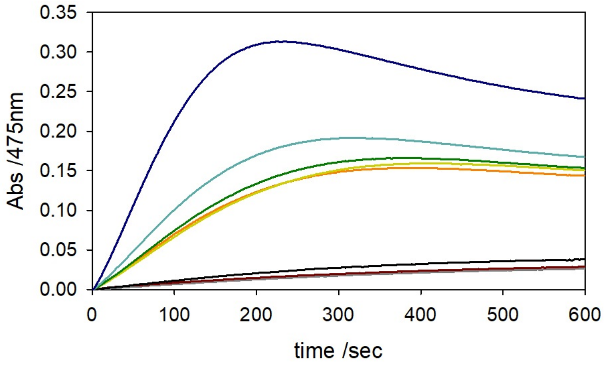

Figure S11. Kinetic profile of DA (3 mM) oxidation with time in 50 mM phosphate buffer solution at pH 7.4 and 37 °C in presence of hemin (2 μM) (brown trace) or hydrogen peroxide (2.5 mM, black), after the addition of both hemin and peroxide (orange) and upon the addition of Aβ_{16} at 2 μM (light green), 10 μM (green), 40 μM (light blue) and 200 μM (blue) as determined by monitoring the absorption intensity at 475 nm as a function of time. The autoxidation trace is shown as grey curve.

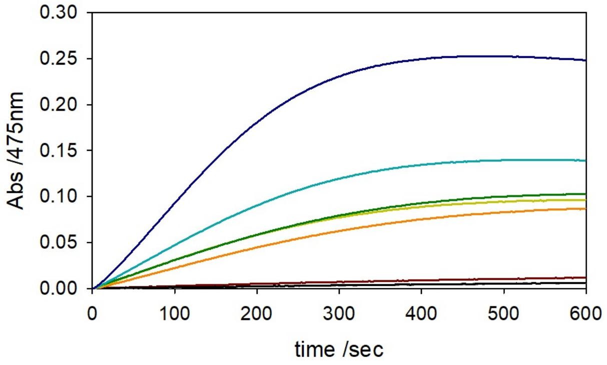

Figure S12. Kinetic profile of DA (3 mM) oxidation with time in 50 mM phosphate buffer solution at pH 6.3 and 37 °C in presence of hemin (2 μM) (brown trace) or hydrogen peroxide (2.5 mM, black), after the addition of both hemin and peroxide (orange) and upon the addition of Aβ_{16} 2 μM (light green), 10 μM (green), 40 μM (light blue) and 200 μM (blue) as determined by monitoring the absorption intensity at 475 nm as a function of time. The autoxidation trace is shown as grey curve.

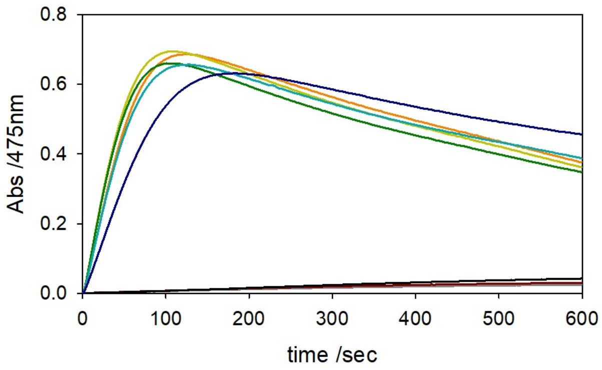

Figure S13. Kinetic profile of DA (3 mM) oxidation with time in 50 mM phosphate buffer solution at pH 6.3 and 37 °C in presence of hemin-glycyl-L-histidine methyl ester (hemin-GH) (2 μM) (brown trace) or hydrogen peroxide (2.5 mM, black), after the addition of both hemin-GH and hydrogen peroxide (orange) and upon the addition of 2 μM (light green), 10 μM (green), 40 μM (light blue) and 200 μM Aβ_{16} (blue) as determined by monitoring the absorption intensity at 475 nm as a function of time. The autoxidation trace is shown as a grey curve.

Table S1. Kinetic constants for the oxidation of DA (λ = 470 nm; ε = 3300 $M^{-1}cm^{-1}$)d by hydrogen peroxide and hemin-glycyl-L-histidine methyl ester (hemin-GH) with varying amounts of $A\beta_{16}$ according to the following conditions: hemin-GH (2 μM), DA (3 mM), H_2O_2 (2.5 mM) in pH 6.3 phosphate buffer solution (50 mM) at 37 °C. The calculated variability for all cases was equal to or less than 0.001 s^{-1} as based on at least 2 repeat measurements

.

Species	k_{obs} [s^{-1}]
Hemin-GH	0.785
$A\beta_{16}$ (1 equiv.)	0.819
$A\beta_{16}$ (5 equiv.)	0.824
$A\beta_{16}$ (20 equiv.)	0.773
$A\beta_{16}$ (100 equiv.)	0.501

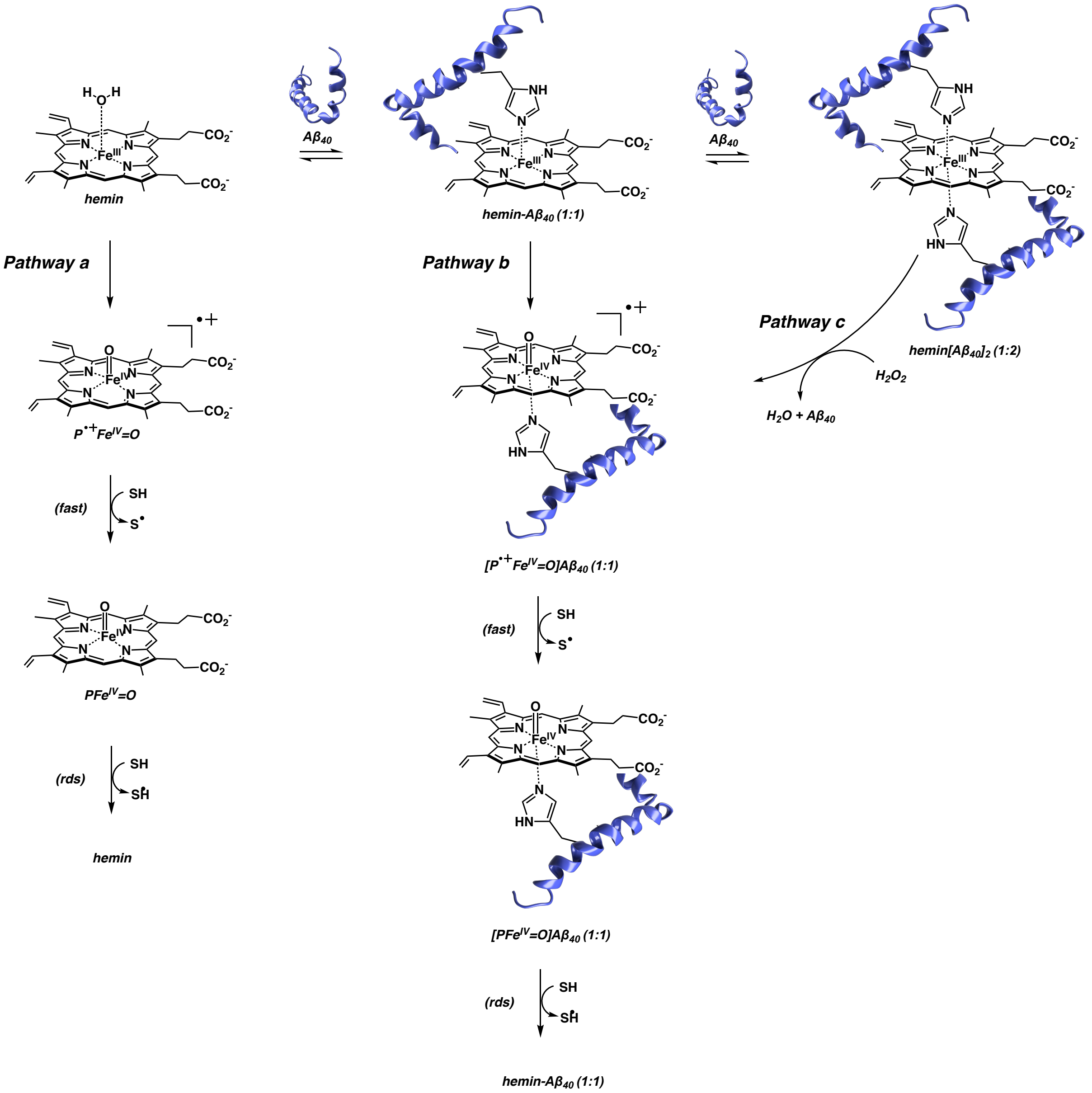

Scheme S2. Proposed reaction mechanism explaining the observed peroxidase-like reactivity of hemin-$A\beta_{40}$ complexes. K_1 and K_2 are the equilibrium constants for heme binding to one and two equivalents of $A\beta_{40}$ or $A\beta_{16}$, respectively, and SH is a generic substrate. Path *a*, path *b*, and path *c* indicate the reaction mechanism of H_2O_2 with free hemin, [hemin(Aβ)] complex and [hemin($A\beta)_2$] complex, respectively.

Table S2. Modification of Aβ16 peptide as inferred from HPLC-MS analysis observed upon reaction of hemin (6 μM), Aβ16 (30 μM), hydrogen peroxide (1 mM) and DA (3 mM) in pH 7.4 phosphate buffer solution (50 mM) at 37 °C.

Time (min)	Aβ16	+16*	+32	+48	+147	+96	+80	Dimer	Fragments
**30**	**79%**	1%	1%	1%	-	6%	4%	2%	6%
**120**	**79%**	2%	1%	2%	-	8%	4%	2%	2%

*O-atom insertion is believed to occur on H13

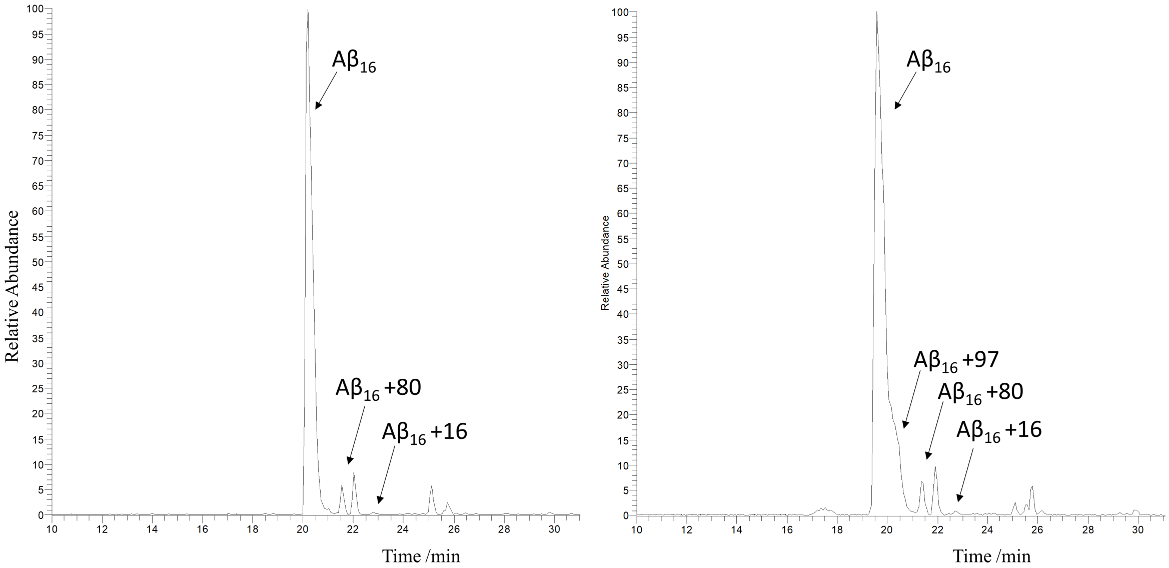

Figure S14. HPLC-MS elution profiles of Aβ16 (30 μM) in phosphate buffer solution (50 mM) pH 7.4 in the presence of hemin (6 μM), H_2O_2 (1 mM) and DA (3 mM) after 30 min (left panel) and 120 min (right panel) reaction time at 37 °C.

Table S3. Modification of Aβ16 peptide as inferred from HPLC-MS analysis upon reaction of hemin (6 μM), Aβ16 (30 μM) and hydrogen peroxide (1 mM) in phosphate buffer solution (50 mM) pH 7.4 at 37 °C.

Time (min)	Aβ16	+16*	+32	+48	+96	+80	Dimer	Fragments
**30**	**85%**	3%	-	-	1%	9%	2%	-
**120**	**84%**	3%	2%	1%	3%	3%	3%	1%

*The O-atom arises from oxidative modification of Y10 or H13
